# Human TREX component Thoc5 affects alternative polyadenylation site choice by recruiting mammalian cleavage factor I

**DOI:** 10.1093/nar/gkt414

**Published:** 2013-05-17

**Authors:** Jun Katahira, Daisuke Okuzaki, Hitomi Inoue, Yoshihiro Yoneda, Kazumitsu Maehara, Yasuyuki Ohkawa

**Affiliations:** ^1^Biomolecular Networks Laboratories, Graduate School of Frontier Biosciences, Osaka University, 1-3 Yamadaoka, Suita, Osaka 565-0871, Japan, ^2^Department of Biochemistry, Graduate School of Medicine, Osaka University, 2-2 Yamadaoka, Suita, Osaka 565-0871, Japan, ^3^DNA-Chip Developmental Center for Infectious Diseases, Research Institute for Microbial Diseases, Osaka University, 3-1 Yamadaoka, Suita, Osaka 565-0871, Japan and ^4^Department of Advanced Medical Initiatives, Division of Epigenetics, Faculty of Medicine, Kyushu University, 3-1-1 Maidashi, Higashi-ku, Fukuoka 812-8582, Japan

## Abstract

The transcription-export complex (TREX) couples mRNA transcription, processing and nuclear export. We found that CFIm68, a large subunit of a heterotetrameric protein complex mammalian cleavage factor I (CFIm), which is implicated in alternative polyadenylation site choice, co-purified with Thoc5, a component of human TREX. Immunoprecipitation using antibodies against different components of TREX indicated that most likely both complexes interact via an interaction between Thoc5 and CFIm68. Microarray analysis using human HeLa cells revealed that a subset of genes was differentially expressed on Thoc5 knockdown. Notably, the depletion of Thoc5 selectively attenuated the expression of mRNAs polyadenylated at distal, but not proximal, polyadenylation sites, which phenocopied the depletion of CFIm68. Chromatin immunoprecipitation coupled with high-throughput sequencing (ChIP-Seq) indicated that CFIm68 preferentially associated with the 5′ regions of genes; strikingly, the 5′ peak of CFIm68 was significantly and globally reduced on Thoc5 knockdown. We suggest a model in which human Thoc5 controls polyadenylation site choice through the co-transcriptional loading of CFIm68 onto target genes.

## INTRODUCTION

In the nucleus of eukaryotic cells, precursor mRNAs (pre-mRNAs) undergo a series of processing steps that include capping at the 5′-end, splicing and cleavage/polyadenylation at the 3′-end, thereby acquiring full maturity and export/translation competency. Although most of these steps can be reconstituted separately as individual *in vitro* reactions, these processes are inter-dependent and streamlined through the cooperation of the transcription machinery with *trans*-acting factors *in vivo*. However, failures in mRNA processing result in the formation of defective messenger ribonucleoproteins (mRNPs), which are confined to the nucleus and eventually eliminated through mRNA surveillance mechanisms (1–5).

Efficient mRNA 3′-end formation is coupled to transcription termination, the release of the transcripts from genetic loci and subsequent nuclear export of mature transcripts. This process also affects the quality of the mature mRNAs as templates for protein translation (4,6,7). Defects in mRNA 3′-end formation result in the accumulation of mRNAs at nuclear transcription foci in different organisms (8,9). In mammals, cleavage and polyadenylation require *cis*-acting signals on pre-mRNAs, such as the A(A/U)UAAA hexamer, U- or UG-rich downstream elements and additional accessory elements (10,11). Canonical cleavage and polyadenylation factors, including the mammalian cleavage factors I and II (CFIm and CFIIm), cleavage and polyadenylation specificity factor (CPSF) and cleavage stimulating factor (CstF), recognize the *cis*-signals and catalyze endonucleolytic cleavage. CFIm, which is a heterotetrameric complex composed of two small and large subunits (12), seems to be unique for metazoan species and is essential for pre-mRNA cleavage *in vitro* (13–15). Poly(A) polymerase, in association with poly(A)-binding protein II, subsequently adds a polyadenylate tail to the 5′-cleavage product. The recruitment of pre-mRNA 3′-end processing factors occurs co-transcriptionally through direct and indirect interactions with RNA polymerase II (RNAPII) (1,2,4,5,16).

The yeast transcription-export complex (TREX), which is composed of the heterotetrameric THO complex, the adaptor mRNA-binding protein Yra1, a DEAD-box–type RNA helicase Sub2 and the SR-like proteins Gbp2 and Hrb1, and Tex1 plays a central role in coupling of the transcription and nuclear export of mRNAs (17–22). Mutations in the TREX components result in the nuclear accumulation of bulk poly(A)^+^ RNAs (23). Yeast TREX, which is co-transcriptionally recruited to active genes, facilitates the loading of a subset of proteins to nascent transcripts and the formation of functional mRNPs (24,25). Recent data also indicate that a transcription elongation factor stabilizes TREX occupancy at transcribed genes (26). Biochemical and genetic analyses in yeast have unveiled the molecular mechanism of the TREX function. In TREX mutants, the *HSP104* mRNA is retained at or in close proximity to the transcription site and destabilized because of poor polyadenylation activity (9,27,28). The yeast TREX components also exhibit extensive genetic and physical interactions with pre-mRNA 3′-end processing factors (28–30). Moreover, the depletion of Yra1 results in the precocious recruitment of Clp1, a yeast CF1 component, to target pre-mRNAs, perturbing normal polyadenylation site choice (31). Thus, the function of yeast TREX has a close connection with pre-mRNA 3′-end formation.

Evolutionarily conserved TREX has also been identified in metazoan species. It comprises the heterohexameric THO complex, Aly and Uap56 in mammals and fruit flies. The metazoan THO complex contains several unique components, such as Thoc5 and Thoc6; direct counterparts of these factors have not been identified in *Saccharomyces cerevisiae* (32–34). The involvement of metazoan TREX in bulk poly(A)^+^ RNA export remains controversial (35,36). Microarray-based genome-wide analyses have revealed that in fruit flies and mice, TREX is engaged in the nuclear export of only a subset of mRNAs, including heat shock mRNAs (32,37). Although the molecular functions of metazoan TREX have not been fully elucidated, 3′-end cleavage of the *HSP70* pre-mRNA is reportedly impeded on knockdown of the THO components in *Drosophila* (38). Moreover, the accumulation of *HSP70* mRNA at nuclear transcription foci was detected in TREX-depleted human cells (39). Taken together, these data suggest that metazoan TREX might also play roles in pre-mRNA 3′-end formation, similar to its yeast counterpart.

Here, we demonstrate that human THO/TREX interacts with the pre-mRNA cleavage factor CFIm68. In addition, DNA microarray-based gene expression analysis in Thoc5-depleted cells revealed that the expression of at least hundreds of non-heat shock genes is under the control of Thoc5. Strikingly, on depletion of Thoc5, the polyadenylation sites of target genes shifted toward proximal; thus, the expression of mRNA species with longer 3′-UTRs was selectively diminished. Similarly, the knockdown of CFIm68 resulted in the selective repression of mRNAs with longer 3′-untranslated regions (UTRs) as previously reported (40). Chromatin immunoprecipitation (ChIP) analysis indicated that knockdown of Thoc5 reduces the association of CFIm68 with the 5′ regions of genes. From these data, we propose a model in which human Thoc5 is required for the co-transcriptional recruitment of CFIm68 to active genes and enables the utilization of distal alternative polyadenylation sites.

## MATERIALS AND METHODS

### Reagents

**Figure 1.**
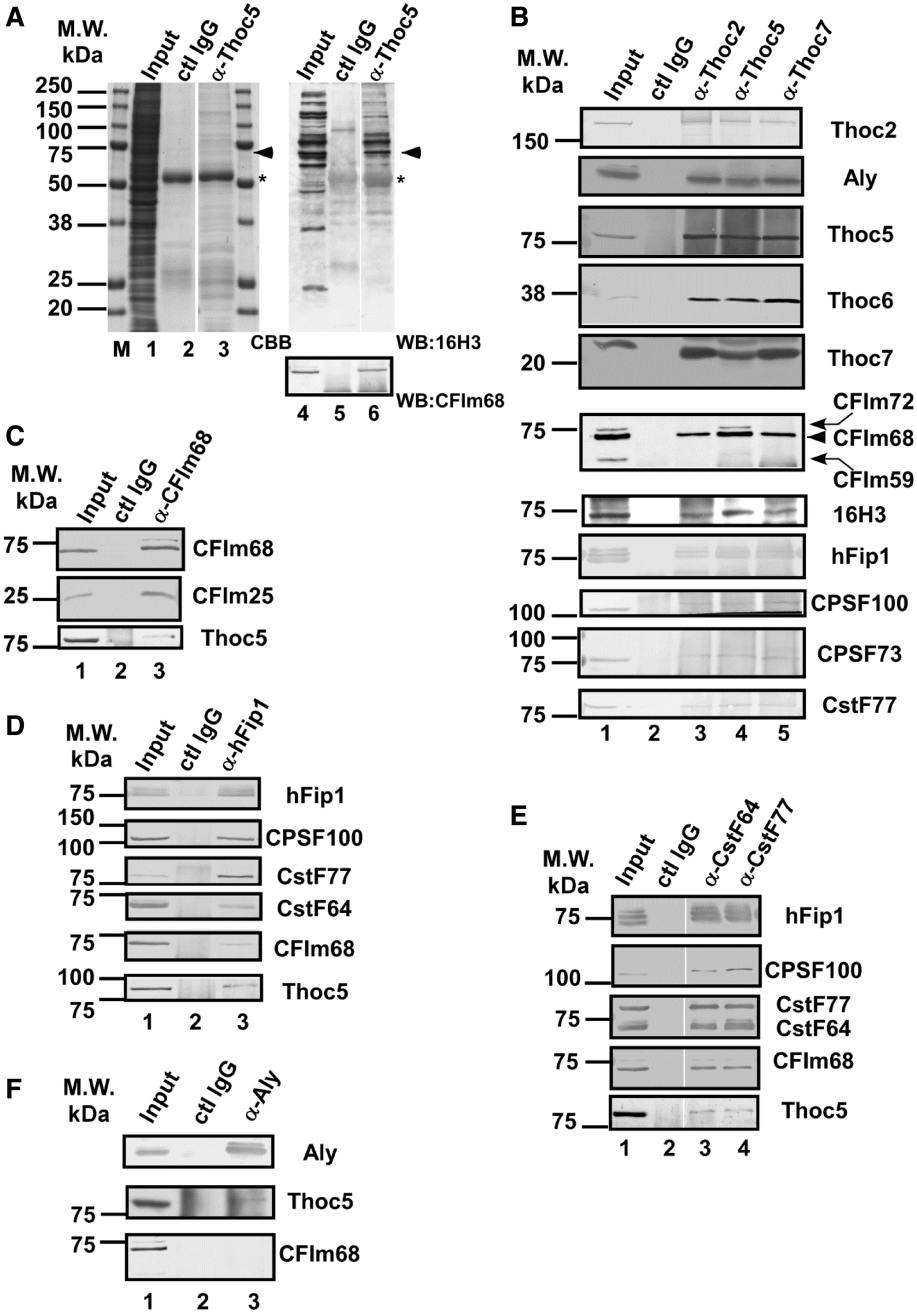
Thoc5 interacts with pre-mRNA cleavage and polyadenylation factors. (**A–F**) RNase A-treated HeLa whole-cell extract was subjected to immunoprecipitation using the antibodies indicated on top of each lane. (**A**) The immune pellets were subjected to SDS–PAGE, followed by CBB staining (left) or western blotting using the indicated antibodies (right). The position of the 70-kDa protein is indicated with arrowheads. The asterisks indicate the position of IgG heavy chain. (B–F) The immune pellets were subjected to western blotting using the antibodies indicated at the right of each panel. In (B), the position of CFIm68 is indicated with an arrowhead. The anti-CFIm68 antibody also cross-reacts with CFIm72 and CFIm59 (indicated with curved arrows), which are detected as slower and faster migrating bands, respectively (14).

Antibodies against the human THO/TREX components and Tap have been previously described (39,41). Rabbit polyclonal antibodies against CFIm68 and CFIm25 (14) were kindly provided by Dr Walter Keller and Dr Georges Martin. Anti-SR (16H3, Zymed) (42), anti-CPSF68 (CFIm68), anti-CPSF73, anti-CstF64, anti-CstF77, anti-hFip1 (Bethyl Laboratories), anti-mouse IgG (Zymed or Rockland), anti-CPSF100 (Sigma), anti-β-actin (AC-15, Sigma), anti-Thoc6 (Abnova), anti-Aly (11G5, Abcam) and normal mouse IgG (Santa Cruz) antibodies were commercially acquired. Horseradish peroxidase- or alkaline phosphatase-conjugated secondary antibodies were purchased from Bio-Rad. In Figure 1B, the large subunits of CFIm were detected by using the ReliaBLOT reagent (Bethyl Laboratories). Protein A-conjugated Sepharose and GammaBind Plus Sepharose were obtained from Sigma and GE healthcare, respectively.

### Immunoprecipitation

HeLa cells grown in 10-cm dishes (1 dish per immunoprecipitation) were harvested, resuspended in RSBN [10 mM Tris–HCl (pH 7.4)/100 mM NaCl/2.5 mM MgCl_2_/0.1% NP-40] containing 40 µg/ml RNase A and lysed by brief sonication. After centrifugation at 12 000 rpm for 10 min, the soluble supernatant was mixed with protein A-conjugated Sepharose beads that had been incubated with various antibodies. Rabbit anti-mouse IgG antibodies were used as a control. For Aly immunoprecipitation, GammaBind Plus Sepharose was used instead of protein A-conjugated Sepharose and normal mouse IgG was used as control. After incubation at 4°C for 3 h, the beads were washed five times with RSBN, and the bound proteins were eluted by boiling in sodium dodecyl sulfate–polyacrylamide gel electrophoresis (SDS–PAGE) sample buffer.

### Microarray analysis

A summary of the microarray analysis is shown in Supplementary Figure S1A. Two independent experiments using different siRNAs against Thoc5 (siThoc5-1 for experiment 1 and siThoc5-2 for experiment 2) and CFIm68 (siCFIm68-1 for experiment 1 and siCFIm68-2 for experiment 2) were performed. In both experiments, siDsRed was used as the negative control. HeLa cells were treated with the siRNAs for 72 h. Total RNAs with a high-RNA integrity value (RIN = 9) were isolated from each siRNA-treated culture using the TRIzol reagent (Invitrogen). The RNA samples (100 ng) were reverse transcribed with T7-oligo(dT), and biotinylated anti-sense RNA was synthesized using the GeneChip 3′ IVT Express kit (Affymetrix). Labeled anti-sense RNA was hybridized to the Human Genome U133 Plus 2.0 array (Affymetrix). The scanned images were analyzed using Affymetrix GCOS software and processed by MAS5.0 normalization to obtain the signal intensity and detection call (present, marginal or absent) for each probe set. After normalization, the data were log_2_-transformed and processed using a 75-percentile shift. For gene expression analysis, the data from the two experiments were averaged, and the insignificant probe data were discarded. The statistical significance of the differentially expressed genes was determined using Student’s *t*-test. Subio Platform and Subio Basic Plug-in (version 1.11; Subio, www.subio.jp) were used to calculate the between-sample average fold-change and for data mining. The NextBio software (43) was used to analyze the correlation between the Thoc5- and CFIm68-knockdown experiments. The genes that were significantly downregulated (at least 2-fold downregulation, Student’s *t*-test *P* < 0.2) by siThoc5 treatment were identified as ‘Thoc5 target candidate genes’ (317 genes). The array data are available from the Gene Expression Omnibus (GEO; www.ncbi.nlm.nih.gov/geo) under the accession number GSE42327.

The distal/proximal ratios were calculated using the following method. The probe sets targeting the most 5′-regions of the last exon of a specific gene were identified and defined as ‘proximal probes’ (e.g. the probe set ‘214513_s_at’ shown in Supplementary Figure S1B). The probe sets targeting more downstream regions (e.g. ‘204312_x_at’ and ‘204313_s_at’ in Supplementary Figure S1B) were defined as ‘distal probes’. The average values obtained at the distal probe positions of each siRNA-treated sample were divided by those of the corresponding proximal probe. The values obtained at each distal probe position of the siThoc5- or siCFIm68-treated samples were further divided by those of the siDsRed-treated sample. The resulting values were designated as ‘distal/proximal ratios’. A distal/proximal ratio of <1 at a distal probe position indicates that the knockdown of either Thoc5 or CFIm68 downregulated the use of the adjacent distal polyadenylation site compared with the control.

All the distal/proximal ratio data for the Thoc5 target candidate genes were collected (630 probe sets). The ‘raw data’ were manually curated (i.e. removal of insignificantly expressed probe data, visual verification of the proximal and distal probe positions and sorting of the data in the proximal to distal order when multiple distal probe data were available). The 289 genes (482 probe sets) identified in this study are listed in Supplementary Table S5.

### Northern blot and ribonuclease protection assay (RPA)

To prepare northern blot probes, cDNA fragments of the *TMED10*, *TIMP2*, *RNMT*, *SUB1*, *RPL22* and *NUDT21* genes were amplified by reverse transcriptase–polymerase chain reaction (RT–PCR) using the primer pairs listed in Supplementary Table S7. The probe for *ACTB* has been previously described (39). The northern blots were performed as previously described (39).

For the RPA probes, genomic DNA fragments near the proximal and distal polyadenylation sites of the *TMED10* and *TIMP2* genes were amplified by PCR using the PCR primer pairs listed in Supplementary Table S6. The amplified genomic DNA fragments were subcloned into the pBluescript SK(−) vector. [^32^P]-labeled anti-sense RNA probes were synthesized using the Riboprobe Systems-T7 kit (Promega) and gel purified. Total RNAs (10 µg) isolated from siRNA-treated HeLa cells were subjected to RPA using the RPA III kit according to the manufacturer’s instruction (Ambion).

### ChIP assays

HeLa cells treated with siThoc5-1 or siDsRed for 72 h were used. HeLa cells grown on 10-cm dishes were treated with 1% formaldehyde for 10 min at room temperature, and the cross-linked cells were washed three times with phosphate-buffered saline and removed from dishes using cell lysis buffer 1 [5 mM 4-(2-Hydroxyethyl)piperazine-1-ethanesulfonic acid (HEPES)–NaOH (pH 7.9)/85 mM KCl/0.5% NP-40]. The cell nuclei were collected by low-speed centrifugation and resuspended in RIPA buffer [10 mM Tris–HCl (pH 8.0)/100 mM NaCl/1 mM ethylenediaminetetraacetic acid (EDTA)/0.5 mM ethylene glycol tetraacetic acid (EGTA)/1% Triton X-100/0.1% deoxycholate/0.05% SDS] supplemented with protease inhibitors (Complete EDTA-free, Roche). The cross-linked chromatin was sheared by sonication (Bioruptor, COSMO BIO), and the insoluble material was removed by centrifugation at 12 000 rpm for 10 min. The supernatant was pre-cleared by incubating with ‘empty’ protein A Sepharose beads for at least 1 h and, then mixed with protein A Sepharose beads pre-incubated with each antibody and salmon sperm DNA (0.625 mg/ml, Invitrogen). For the immunoprecipitation, a mixture of three anti-CFIm68 polyclonal antibodies (1.5 µg each, Bethyl Laboratories) was used. Rabbit anti-mouse IgG (5 µg) was used as the negative control. The mixtures were incubated at 4°C for 16 h. After extensive washing with RIPA buffer, the bound chromatin fragments were released from the beads and de-cross-linked by incubating in 50 mM Tris–HCl (pH 8.0)/5 mM EDTA/1% SDS at 65°C for 12 h. The de-cross-linked chromatin samples were treated with RNase A, followed by de-proteinization by proteinase K treatment and phenol–chloroform extraction. After ethanol precipitation, the ChIP DNA fragments were subjected to PCR and massively parallel sequencing analysis.

### ChIP PCR

The sequences of the primer pairs used for PCR are listed in Supplementary Table S8. Thirty cycles were performed using the Expand Hi-Fi PCR system (Roche), with each cycle consisting of a 30 s denaturation at 95°C, a 30 s annealing at 60°C and a 20 s extension at 70°C. For quantification, the PCR reactions were performed in the presence of [^32^P]-dCTP as a tracer, and the DNA fragments were separated by 5% acrylamide gel electrophoresis. The DNA bands were detected and quantitated using a Bioimaging analyzer (Fuji Film). To monitor specificity, a primer set that amplifies an intergenic region of human chromosome 2 (44) was included. The ChIP efficiency was calculated using the following formula: (V_ChIP_ − V_control_)/V_input_, where V_ChIP_ and V_control_ are the amounts of PCR products obtained using the chromatin fragments immunoprecipitated with specific and control antibodies, respectively, and V_input_ is the amount obtained by using input DNA. Three technical replicates were performed for each PCR. Statistical significance was determined using Student’s *t*-test.

### ChIP-Seq

The ChIP DNA and the input DNA ends were repaired using T4 DNA polymerase, Klenow enzyme and T4 polynucleotide kinase (PNK) (New England Biolabs), followed by treatment with Klenow exo- to add an A base to the 3′-end. After ligation of the Genomic Adaptor Oligo Mix (Illumina) using TaKaRa Ligation Mix (TaKaRa), the adaptor-ligated DNA fragments were amplified with Paired-End Sample Prep Oligo primers (Illumina) for 18 cycles. The amplified library was separated on a 2.0% agarose gel, and the samples were purified using the QIAquick MinElute kit (Qiagen) after each preparation step. The purified library was used for cluster generation and sequencing analysis using the Genome Analyzer GAIIx (Illumina). The raw Illumina sequencing data are available from the DNA Data Bank of Japan (DDBJ) (DDBJ accession number: DRA000863).

### ChIP-Seq data analysis

The sequence reads for CFIm68, CstF64, Xrn2 and the input were aligned to the human genome (hg19) using the Bowtie software (parameter: -v 3 –m 1). The MACS software (ver. 1.4.1) was used for peak detection and identification of the binding sites of CFIm68 (45,46). The parameters for MACS were ‘-bw 600’, and the other parameters were the software’s default. To detect the binding site of CFIm68, the lengths of all human genes were normalized to 1, and the percentages of the detected peaks were calculated from 0 (transcription start site: TSS) to 1 (transcription termination site: TTS) for every width of 0.01 on all genes (Figure 4A and Supplementary Figure S3). We used 20 374 human genes as the total number of genes for which a definition was obtained from UCSC’s ‘knownGene’ table. For Supplementary Figure S3A, the peak information was obtained from the ENCODE project (TBP: wgEncodeEH001790, Ser2-P RNAPII: wgEncodeEH001838) (47). The details of the CstF64 and Xrn2 ChIP-seq data will be described elsewhere.

RPM (reads per million reads) (48) of proximal promoter regions (<2 kb from TSS) was calculated on all gene. The dRPM scores were defined as difference of RPM between ChIP and input samples and represent deviations of ChIP signal from input signal.

### Miscellaneous

Western blotting and siRNA treatment of HeLa cells were performed as described previously (39). The sequences of siThoc5-1, siThoc5-2 and siDsRed have been described (39). The sequences of siCFIm68-1 and siCFIm68-2 are listed in Supplementary Table S9.

## RESULTS

### Thoc5 interacts with the pre-mRNA cleavage and polyadenylation factor CFIm68

We performed immunoprecipitation assays to identify protein factors that interact with Thoc5, a component of human TREX. As shown in Figure 1A, a protein with an apparent molecular weight of 70 kDa was reproducibly co-immunoprecipitated with an antibody raised against Thoc5 (39). The 70-kDa protein contained an epitope shared with a subset of SR family proteins that was recognized by the monoclonal antibody 16H3 (42) (Figure 1A, lane 6). Among the proteins recognized by this antibody, we noted that CFIm68, a component of mammalian cleavage factor I (CFIm), migrated within a similar molecular weight range (14). As expected, the 70-kDa protein was recognized by an anti-CFIm68 antibody (14) (Figure 1A). Because CFIm is a component of the large pre-mRNA 3′-end processing machinery (10,11), we examined whether other pre-mRNA cleavage and polyadenylation factors co-purified with Thoc5. As shown in Figure 1B, CPSF100, hFip1, CPSF73 and CstF77 were also co-immunoprecipitated with Thoc5. In reciprocal immunoprecipitation experiments in which the components of the cleavage and polyadenylation factors were pulled down using specific antibodies, Thoc5 was recovered in the immune pellets, albeit less efficiently (Figure 1C and D). We observed that pre-mRNA cleavage and polyadenylation factors were also co-precipitated with anti-Thoc2 and anti-Thoc7 antibodies (Figure 1B). Notably, however, less CFIm68 co-purified with Thoc2 and Thoc7 than with Thoc5, although the amounts of the co-purified THO/TREX components were similar (Figure 1B). In addition, among the different large subunits of CFIm (14), CFIm68 and CFIm72, but not CFIm59, were preferentially co-precipitated by the anti-Thoc5 antibody (Figure 1B). In addition, CFIm68 did not efficiently co-purify with Aly (Figure 1F). Taken together, we concluded that most probably the THO complex interacts with CFIm through the interaction between Thoc5 and CFIm68.

### Identification of human genes regulated by Thoc5

**Figure 2.**
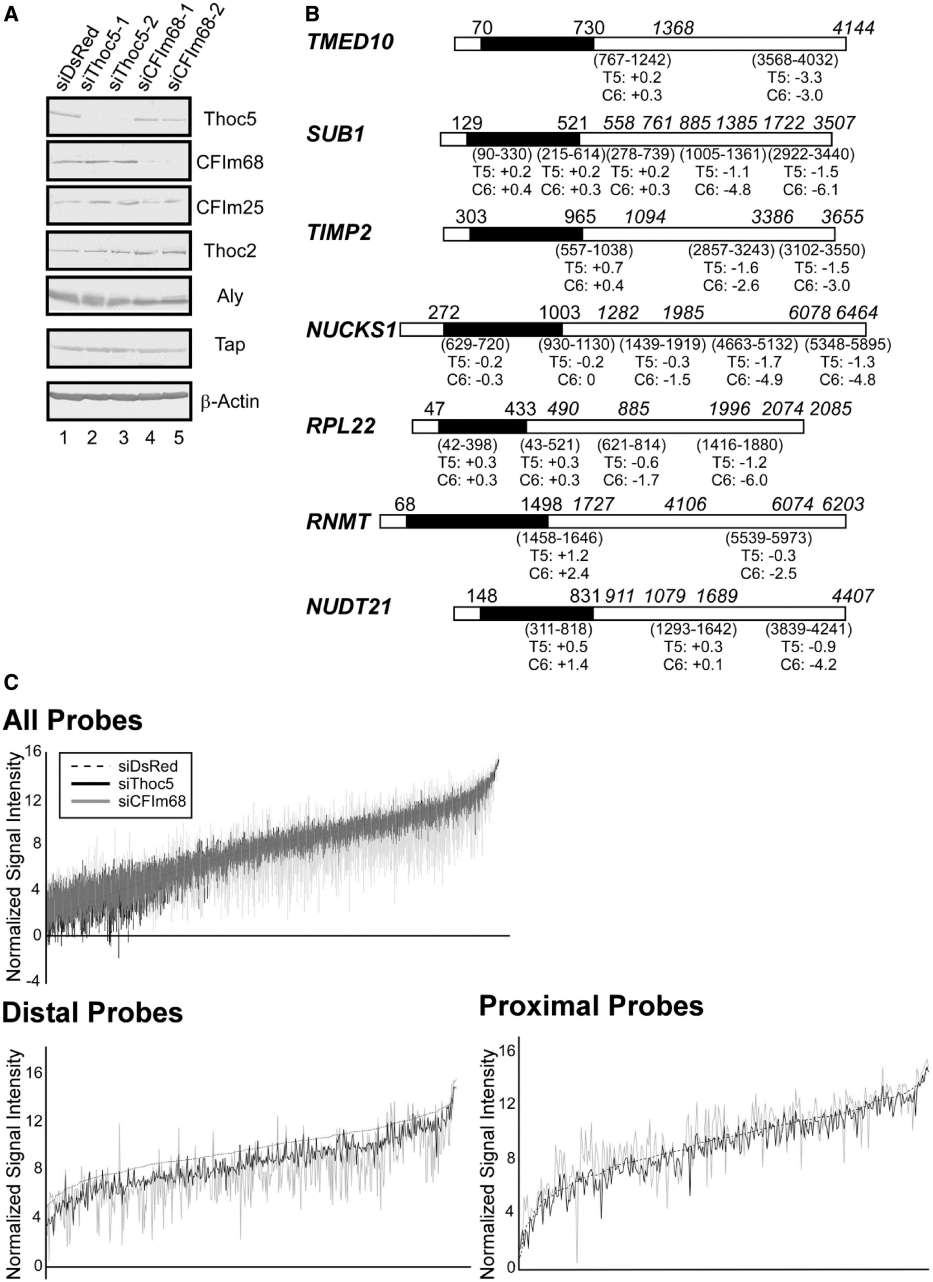
Identification of Thoc5-regulated genes. (**A**) HeLa cells were treated with the indicated siRNAs for 72 h. Whole-cell extracts prepared from the siRNA-treated cells were subjected to western blotting using the indicated antibodies. (**B**) The structures of the *TMED10* (NM_006827), *SUB1* (NM_006713), *TIMP2* (NM_003255), *NUCKS1* (NM_022731), *RPL22* (NM_000983), *RNMT* (NM_003799) and *NUDT21* (NM_007006) mRNAs, which show a typical alternative polyadenylation pattern (i.e. downregulated more severely at the distal probe positions), are shown. The black and white boxes indicate the coding and untranslated regions, respectively. The numbers above the black boxes indicate the positions of the translation start and stop codons. The positions of the polyadenylation sites (either obtained from the NCBI Refseq sequence database or deduced from different EST clone sequences) are italicized and shown above the boxes. The array probe positions are given in parentheses below each box. The log_2_-fold changes at each probe position are also shown (T5, siThoc5-1; C6, siCFIm68-1-treated cells; +, upregulated; −, downregulated). Note that the structures of the mRNAs, the probe positions and polyadenylation sites are not drawn to scale. (**C**) The signal intensity (*y*-axis, log_2_-transformed) at all significantly expressed (upper panel, 11 216 probe sets), distal (lower left panel, 482 probe sets) and proximal (lower right panel, 289 probe sets) probe positions (*x*-axis) listed in Supplementary Table S5 is shown. The data for the siDsRed-treated sample (dashed line) at each probe position are plotted in ascending order, and those of the siThoc5- (black line) and siCFIm68-treated (gray line) samples at the corresponding probe positions are overlaid. Note that the signals for the distal probe sets were reduced on Thoc5 and CFIm68 depletion.

Heat shock genes have been extensively characterized as the targets of metazoan THO/TREX (32,38,39,49). To expand the list of the target genes of human THO/TREX, we performed a genome-wide gene expression analysis in Thoc5- and CFIm68-depleted HeLa cells using an expression microarray (Supplementary Figure S1 and ‘Materials and Methods’ section). The siRNAs efficiently targeted Thoc5 and CFIm68, with negligible effects on non-targets (Figure 2A). Treatment with either of the two siRNAs against Thoc5 resulted in >2-fold downregulation of expression at the 1048 (siThoc5-1) and 1087 (siThoc5-2) probe positions. By contrast, the CFIm68 siRNAs strongly impacted the expression of genes, and significant downregulation was observed at the 3828 and 3486 probe positions by siCFIm68-1 and siCFIm68-2 treatment, respectively (Supplementary Figure S2A). After normalizing the data from the two independent experiments, we found that only a subset of genes was differentially expressed under Thoc5-depleted condition (Supplementary Figure S2B, Bioset1). Moreover, we also noted that the majority of the probe sets that were commonly dysregulated under the Thoc5- and CFIm68-depleted conditions exhibited strong positive correlations (Supplementary Figure S2B, lower panel).

To avoid the selection of off-targets, only the 289 genes (482 probe sets) that were significantly downregulated in both experiments and passed our data quality control (see ‘Materials and Methods’ section) were selected as candidate human Thoc5 target genes. The expression of these genes was then further analyzed. Interestingly, CFIm, of which the CFIm68 large subunit was identified as a novel Thoc5 interacting factor, has been implicated in alternative polyadenylation site choice *in vivo* (40,50). The 3′ expression array used in this study contains multiple probe sets that could interrogate the expression of mRNA species with different 3′-ends, enabling the monitoring of alternative polyadenylation events (51,52). Therefore, we further analyzed the array data to evaluate the usage of different polyadenylation sites under Thoc5-depleted conditions (for experimental details, see Supplementary Figure S1 and ‘Materials and Methods’ section). Strikingly, a closer examination of the expression of the 289 genes at different probe positions revealed that 275 genes (95%) were selectively downregulated at distal positions (Figure 2B and C; Supplementary Table S5). In contrast, the expression of mRNAs polyadenylated at the proximal positions was either unaffected or increased in many instances (Figure 2B and C and data not shown). Of the 275 genes (416 probe sets), 255 genes (93%) displayed a similar alternative polyadenylation pattern on CFIm68 knockdown (Figure 2B and C and Supplementary Table S5). These data strongly suggest that Thoc5 functions in alternative polyadenylation site choice through the interaction with CFIm68. In most cases, knockdown of CFIm68 resulted in more pronounced and widespread effects on the expression of mRNAs polyadenylated at the distal probe positions (e.g. Figure 2B and C, Supplementary Figure S2B). Therefore, we assumed that rather than being directly involved in the cleavage and polyadenylation reaction, Thoc5 modulates the activity of CFIm through some other mechanism (see later in the text).

### Thoc5 is required for the selection of distal but not proximal polyadenylation sites in target genes

**Figure 3.**
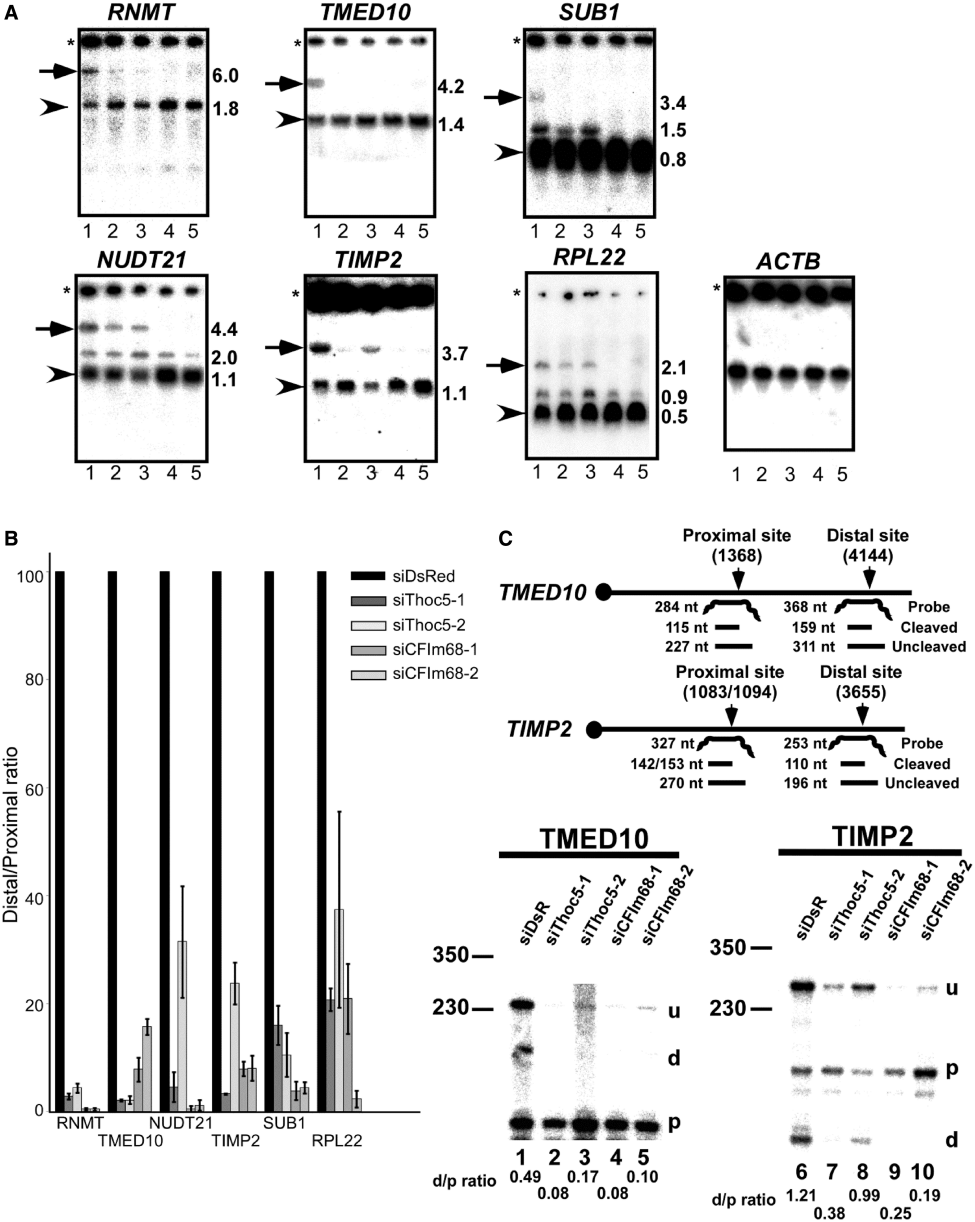
Depletion of Thoc5 and CFIm68 affects polyadenylation site choice. (**A**) Total RNAs prepared from siRNA-treated HeLa cells (Lanes 1, siDsRed; 2, siThoc5-1; 3, siThoc5-2; 4, siCFIm68-1; 5, siCFIm68-2) were subjected to northern blotting using the indicated probes. The transcripts polyadenylated at the most distal (arrows) or proximal (arrowheads) polyadenylation sites are indicated. The approximate transcript sizes are shown to the right of each panel in kilobases. The asterisks indicate the origins of migration. (**B**) The abundance of mRNAs polyadenylated at the most distal and proximal polyadenylation sites was measured by phosphorimaging. The ratios between these mRNA species (distal/proximal ratio) were calculated. The data were converted to per cent of control and are shown as means ±SD of three replicates. (**C**) Upper: The probes used in RPA; the positions of the proximal and distal polyadenylation sites (vertical arrows) and the expected band sizes are shown. Lower: Total RNAs isolated from siRNA-treated HeLa cells were subjected to RPA using mixtures of the TMED10 (left) and TIMP2 (right) probes. The digested probes were separated by electrophoresis in 8 M urea/8% polyacrylamide denaturing gels. The positions of the bands are indicated to the right of each panel (u, uncleaved; d, distal; p, proximal). The ratios between the mRNAs polyadenylated at the distal and proximal sites are shown below each panel (d/p ratio). The positions of the size markers are indicated on the left in bases.

To validate our observations, we selected several genes from the target list and analyzed their expression by northern blotting. In addition, the *RNMT* and *NUDT21* genes, which displayed alternative polyadenylation patterns in human cells (40,50,53) (Figure 2B) but were not listed in Supplementary Table S5, were also analyzed. Under Thoc5- and CFIm68-depleted conditions, the expression of the longer mRNAs of genes (Figure 3A, indicated by arrows) was selectively diminished, confirming the array data. In contrast, the expression of the shortest mRNAs polyadenylated at the most proximal polyadenylation sites was either unchanged or increased (Figure 3A or B). The expression pattern of the *ACTB* gene, which does not harbor alternative polyadenylation sites, was not significantly altered (Figure 3A). Furthermore, mapping of the 3′-ends of the *TMED10* and *TIMP2* mRNAs by RPAs revealed that the use of the distal polyadenylation sites was, indeed, diminished by Thoc5 or CFIm68 depletion (Figure 3C). Based on these data, we concluded that Thoc5, together with CFIm68, functions as a regulator of alternative polyadenylation site choice.

### Recruitment of CFIm68 to the 5′ regions of target genes is diminished by Thoc5 depletion

**Figure 4.**
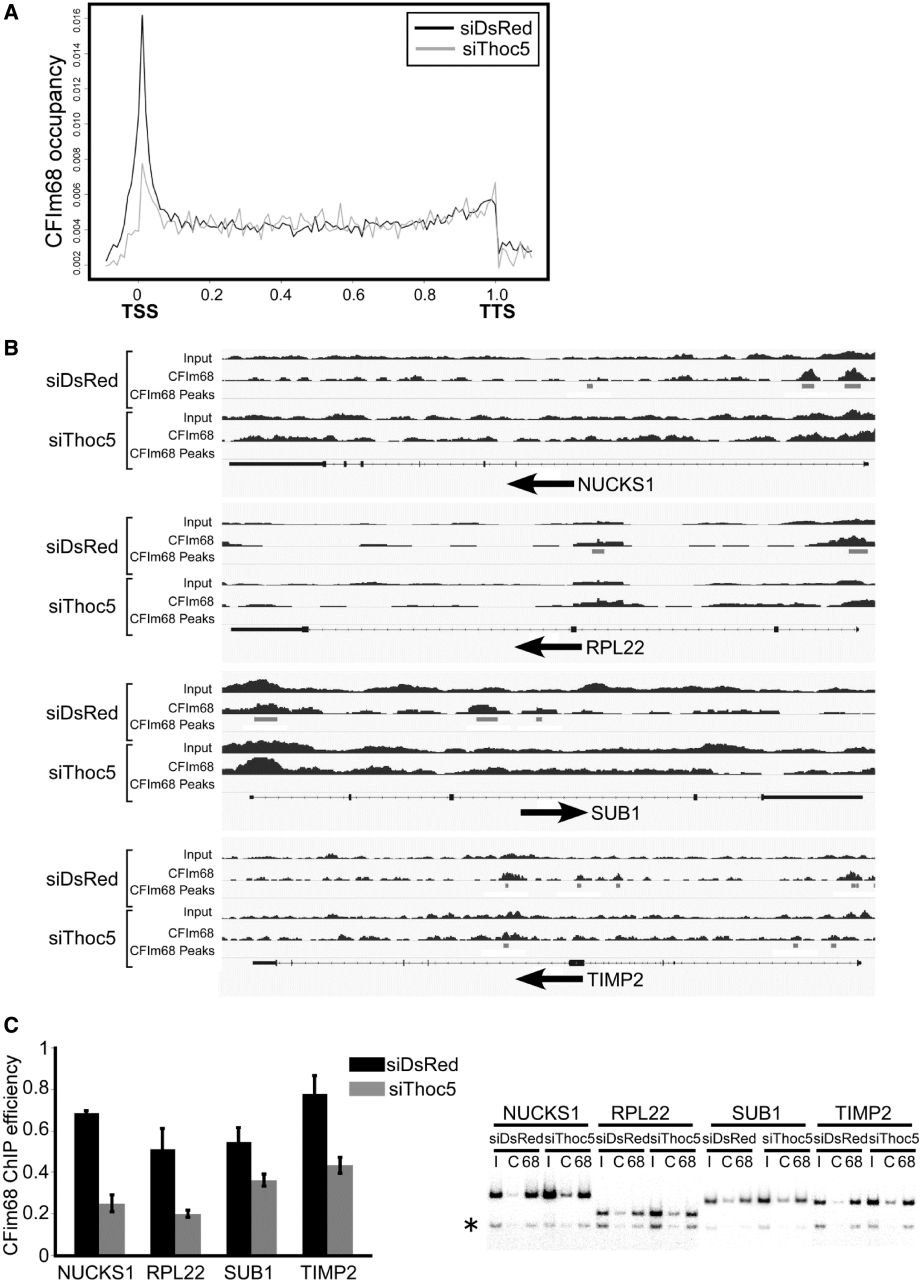
Thoc5 is required for the recruitment of CFIm68 to target genes. (**A**) The distribution of CFIm68 on all annotated human genes. The abundance of CFIm68 binding for the siDsRed- (black line) or siThoc5- (gray line) treated samples is plotted along the length of the genes. The lengths of all annotated genes are scaled to 1; thus, the transcription start sites (TSSs) and transcription termination sites (TTSs) are set at 0 and 1, respectively (*x*-axis). Note that the accumulation of CFIm68 at the 5′-regions of the genes was significantly reduced on knockdown of Thoc5. (**B**) Distribution of CFIm68 on selected Thoc5 target genes under control and Thoc5-depleted conditions. The numbers of ChIP-seq reads are plotted on the genomic regions (black bar charts). Peaks of CFIm68 binding identified by the MACS peak-finding algorithm are indicated by gray horizontal bars (CFIm68 peaks). The gene structures are indicated at the bottom of each graph. The horizontal arrows below the gene structures indicate the orientations of the genes. The boxes indicate exons and the thin lines indicate introns. The protein-coding regions are indicated by thicker boxes. Note that the CFIm68 peaks at the 5′ regions of the genes disappear on Thoc5 depletion. (**C**) HeLa cells treated with siDsRed and siThoc5-1 were subjected to ChIP assays using a mixture of anti-CFIm68 antibodies. PCR amplification of the 5′-region of the indicated genes was performed in the presence of [^32^P]-dCTP. The PCR products were separated by polyacrylamide gel electrophoresis. A Bioimaging analyzer was used for visualization and quantification. Left: The ChIP efficiency (IPed/Input) was calculated as explained in ‘Materials and Methods’ section and is presented as the means ±SD; the significant differences were analyzed by Student’s *t*-test (*P* values, *NUCKS1*, 5.17 × 10^−5^; *RPL22*, 0.00583; *SUB1*, 0.01456; *TIMP2*, 0.00332). Right: Representative PCR reactions are shown. I, input; C, immunoprecipitation with control antibody; 68, immunoprecipitation with the anti-CFIm68 antibody. The asterisk indicates the position of the intergenic control.

Similar to other pre-mRNA cleavage and polyadenylation factors, CFIm is also co-transcriptionally recruited to genes (15). To determine whether Thoc5 regulates the efficiency of CFIm68 recruitment to genes, we performed a genome-wide ChIP-Seq analysis in which HeLa cells treated with siThoc5 or control siDsRed were subjected to ChIP using anti-CFIm68 antibodies. Each immunoprecipitate was de-cross-linked, de-proteinized and subjected to massively parallel sequencing using the Illumina Platform. Cross-linked chromatin samples that were not subjected to immunoprecipitation were treated in the same way and used as input. We obtained 1.4∼2.0 × 10^7^ mappable reads per sample (Supplementary Table S6). In the control siRNA-treated cells, CFIm68 exhibited a sharp, ‘peaky’ accumulation at the 5′ regions of the annotated human genes (Figure 4A, black line). The CFIm68 signal, as well as those of TBP and RNAPII, was enriched around TSS, indicating CFIm68 is recruited to promoter regions (Supplementary Figure S3A). Although the pre-mRNA 3′ processing factors have been shown to exhibit an apparent bimodal localization at both ends of genes (54), the 3′ peak of CFIm68 was not as significant as those of CstF64 and Xrn2 (see Supplementary Figure S3B and C for our own data). Notably, the 5′ peak of CFIm68 decreased significantly and globally on knockdown of Thoc5 by siRNA treatment (Figure 4A, gray line). By examining individual genes, we noted that the averaged dRPM score of the Thoc5 target genes was significantly higher than that of whole-gene in siDsRed-treated cells, whereas the difference was insignificant in siThoc5-treated cells [Z-scores: siDsRed-treated cells, 1.660419 (*P* = 0.04841509); siThoc5-treated cells, 1.103570 (*P* = 0.13488977)]. These data suggest that higher amount of CFIm68 tends to associate with the 5′ regions of the Thoc5 target genes and that the CFIm68 association with the 5′ regions of the Thoc5 target genes is reduced under Thoc5-depleted condition. In fact, the latter observation was consistent with our finding that the CFIm68 peaks at the 5′ regions of Thoc5 target genes, such as *NUCKS1*, *RPL22*, *SUB1* and *TIMP2*, became undetectable under Thoc5-depleted condition (Figure 4B). These findings may also explain the reason why the Thoc5 target genes identified by the microarray analysis exhibit an apparent alternative polyadenylation pattern on Thoc5 knockdown. To evaluate these observations, semi-quantitative PCR was performed, and we observed that the association of CFIm68 with the 5′ regions of the Thoc5 target genes was significantly reduced, when the expression of Thoc5 was knocked down by siRNA treatment (Figure 4C). From these data, we concluded that Thoc5 is required for the recruitment of CFIm68 to active genes, thereby regulating alternative polyadenylation site choice.

## DISCUSSION

Yeast THO/TREX physically and genetically interacts with various mRNA processing factors, including SR-like mRNA-binding proteins (17,18) and pre-mRNA cleavage and polyadenylation factors (28–30). We report here that human THO/TREX interacts with CFIm68, an SR-like component of the metazoan pre-mRNA cleavage and polyadenylation machinery. Heterotetrameric CFIm, which comprises two small subunits (CFIm25) and two large subunits (CFIm68, CFIm72 or CFIm59), binds pre-mRNAs to facilitate the subsequent assembly of CFIIm, CPSF and CstF at the polyadenylation site (12,14,55). Our immunoprecipitation data strongly suggest that the THO and the CFIm complexes most likely interact to each other via an interaction between Thoc5 and CFIm68.

Analysis of the kinetics of *in vitro* cleavage reactions indicates that CFIm acts at one of the earliest steps in the assembly of the cleavage/polyadenylation complex on pre-mRNA (13–15). Although the activity of CFIm is essential for the *in vitro* cleavage reaction (13,14), its depletion did not completely block pre-mRNA cleavage/polyadenylation but induced a systematic use of proximal polyadenylation sites, resulting in preferential expression of mRNAs with shorter 3′-UTRs *in vivo* (40,50). Our data clearly indicate that Thoc5 via its physical interaction with CFIm68 regulates alternative polyadenylation site choice. The ChIP-seq data (Figure 4A) simply indicate the reduction of the number of identifiable CFIm68 peaks at the 5′ regions of genes on Thoc5 depletion. Therefore, we consider that the Thoc5 depletion globally reduces the recruitment of CFIm68 to the 5′ regions of genes. One reason why the targets are restricted to the small subgroup of genes is due to our strict selection of target genes by microarray analysis. In addition, as Thoc5 seems to play only an indirect role in cleavage/polyadenylation reaction (i.e. recruitment of CFIm68, see later in the text), the upstream shift of cleavage/polyadenylation sites by Thoc5 depletion was not as evident and systematic as observed under the CFIm68 depleted condition.

Pre-mRNA cleavage and polyadenylation factors are known to interact with the transcriptional machinery, such that they are co-transcriptionally recruited to pre-mRNAs (1,2,4,5,15,16). However, the molecular mechanisms underlying the recruitment of the individual cleavage and polyadenylation factors have not yet been fully characterized (16). Recently, Johnson *et al.* proposed an elegant model in which Pcf11, a component of yeast CF1A (which also contains the counterpart of the mammalian CFIIm component Clp1), is involved in the recruitment of Yra1, the counterpart of metazoan Aly, to active genes. The dissociation of Yra1 from Pcf11 induces synchronous formation of functional TREX and CF1A on pre-mRNA, thereby coupling transcription, 3′-end formation and the nuclear export of mature mRNA (30). Our data add an additional layer of the co-transcriptional regulation of pre-mRNA cleavage and polyadenylation by the TREX component Thoc5. The association of THO/TREX with multiple cleavage and polyadenylation factors increases the local concentrations of these factors, thereby ensuring efficient pre-mRNA 3′-end processing. Martin *et al.* (40) performed transcriptome-wide mapping of binding sites of 3′-end processing factors and 3′-end processing sites in human HEK293 cells and showed that the 3′ processing factors including CFIm generally associate with the polyadenylation sites most frequently used. The apparent differences in the cross-linked positions of CFIm, as detected by ChIP-seq and PAR-CLIP analyses (e.g. 5′ region versus 3′ region) might indicate the transfer of CFIm from the transcription machinery to nascent transcripts during transcription elongation (see also later in the text).

**Figure 5.**
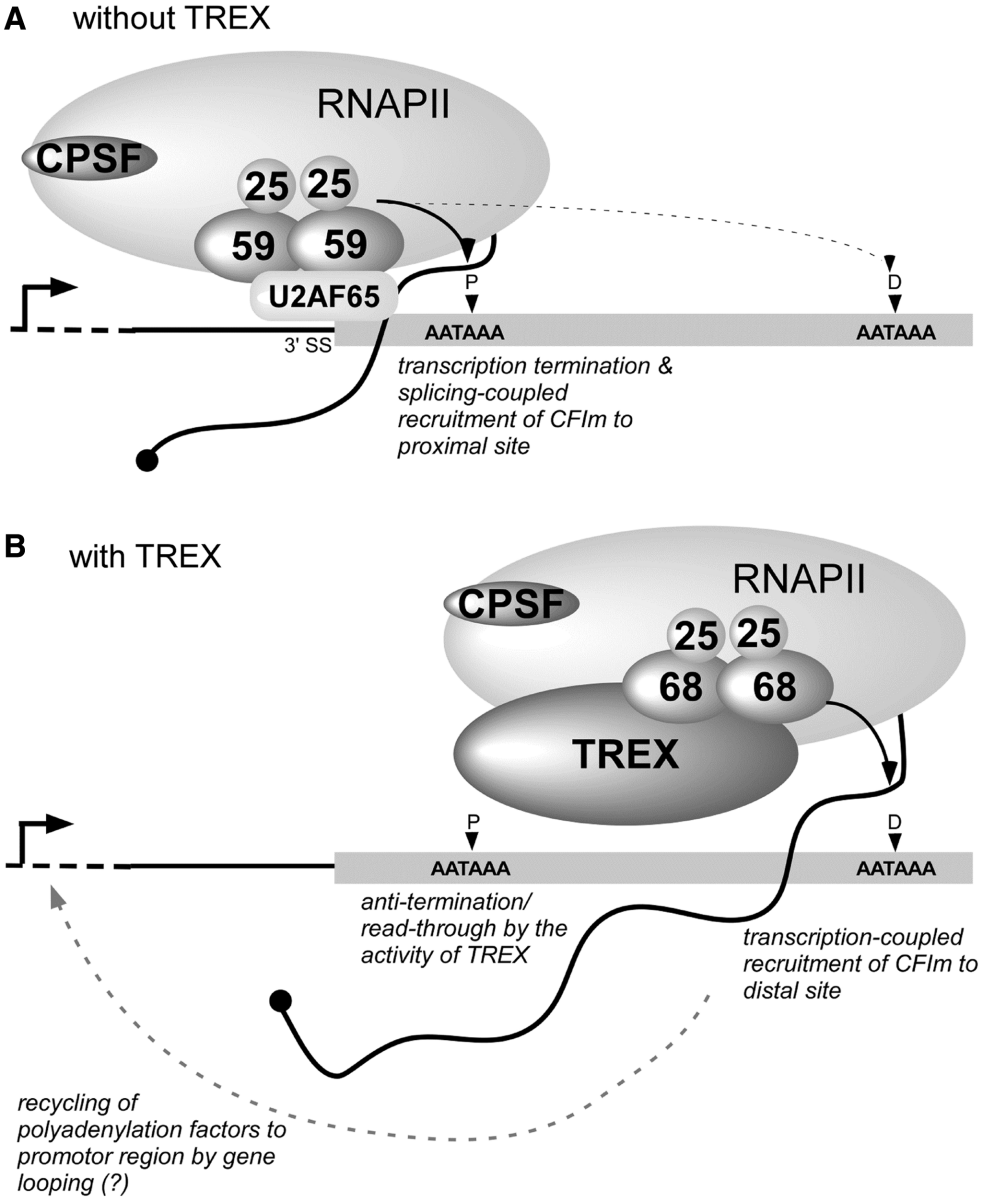
A possible mechanism of THO/TREX-dependent cleavage and polyadenylation at distal polyadenylation sites. The thin line and gray box indicate the last intron and terminal exon, respectively. The curved line with the black circle indicates nascent pre-mRNA. The vertical arrowheads show the different polyadenylation sites in the terminal exon (P, proximal site; D, distal site). The thin dashed line indicates the upstream region of the gene. The vertical line with the horizontal arrow indicates the transcription start site. The ovals with numbers indicate the small and large subunits of CFIm. For simplicity, CFIIm, U2 snRNP, CstF and so forth are not depicted in the diagram. (**A**) CFIm, tethered near the 3′ splice site (3′SS) via the interaction between CFIm59 and the splicing factor U2AF65, triggers the accumulation of cleavage and polyadenylation factors, thereby coupling splicing and polyadenylation (59). The cleavage and polyadenylation machinery formed in this manner may exert a stronger effect on the proximal site (thick arrow) but not on the distal sites (thin dashed arrow). (**B**) In the presence of TREX, a fraction of transcribing RNAPII escapes termination at the proximal site and continues elongating further downstream. CFIm is co-transcriptionally recruited by THO/TREX, thus enabling the cleavage/polyadenylation at the distal site. At present, it is unclear whether two different populations of RNAPII (i.e. with or without associating THO/TREX) exist. After transcription termination at the distal polyadenylation site, CFIm along with other transcription and polyadenylation factors may be recycled back to promoter region by gene looping for other rounds of transcription (61,62).

Pre-mRNA splicing, particularly the removal of the last intron, and 3′-end processing in the terminal exon are mutually coupled (56,57), and this coupling is important for the discrimination of the terminal exon from the internal exons and the delimitation of the 3′-end of a gene (16,58). Among the different large subunits of CFIm, CFIm59 has been proposed to play a specific role in this coupling. The U2 snRNP auxiliary factor U2AF65, which binds to the polypyrimidine tract upstream of the 3′ splice site (3′SS) in the last intron, interacts selectively with CFIm59 but not CFIm68. Through this interaction, U2AF65 facilitates the tethering of CFIm59 to the polyadenylation site downstream of 3′SS (59). In addition, CPSF is recruited to the pre-mRNA through interactions with U2 snRNP (60) (Figure 5A). The majority of the Thoc5 target genes identified in this study harbor a long terminal exon [average length >3000 nt; the average 3′-UTR length of human genes is ∼950 nt (63)] containing multiple polyadenylation sites. On the depletion of either Thoc5 or CFIm68, we observed that mRNAs polyadenylated at distal sites were selectively diminished. In contrast, the expression of the shortest mRNAs polyadenylated at the most proximal site, which is positioned closest to the 3′SS, remained unchanged or even increased in most cases. Indeed, CFIm68 but not CFIm59 was preferentially co-purified with Thoc5 (Figure 1B), and the CFIm68 siRNAs used in this study did not significantly downregulate the expression of CFIm59 (Supplementary Figure S4). These data may support the notion that cleavage/polyadenylation at the proximal sites is mandatory, and those at the distal positions in the terminal exons occur by different mechanisms.

Based on the previous (59) and our observations, we favor a model (Figure 5) in which the polyadenylation at the distal position is mainly coupled with transcription elongation and that Thoc5, most likely as a component of THO/TREX, plays a decisive role in the co-transcriptional recruitment of CFIm68. Probably, THO/TREX (and its associating factors including CFIm itself) facilitates transcription elongation (35) and enables a fraction of RNAPII to read-through the proximal polyadenylation site. At the distal polyadenylation site, CFIm recruited to pre-mRNA expedites subsequent assembly of other cleavage/polyadenylation factors. After cleavage/polyadenylation and transcription termination, CFIm68, as described for RNAPII and other transcription and cleavage/polyadenylation factors, is recycled back to promoter region for other rounds of transcription most probably by gene looping (61,62). It remains unanswered what triggers the transfer of CFIm from THO/TREX to nascent mRNAs and whether and/or how the activity of CFIm is regulated during transcription elongation.

Signals that govern the stability, translatability and intracellular localization of individual mRNAs are often located within the 3′-UTR. The length of 3′-UTRs is globally regulated during development or under different cellular proliferation states by alternative polyadenylation. For example, the 3′-UTRs of mRNAs tend to lengthen progressively during mouse embryonic development (64,65). Transcripts with longer 3′-UTRs could harbor various regulatory elements, such as microRNA-binding sites and stability and localization elements, and the spatio-temporal expression of these transcripts could be more strictly regulated than those with shorter 3′-UTRs (66). One of the Thoc5 targets, the *TIMP2* gene, contains AU-rich elements between the proximal and distal polyadenylation sites. Indeed, the fusion of the longer *TIMP2* 3′-UTR to a luciferase reporter gene significantly reduced the stability of the mRNA (Ryo Shibasaki and J.K. unpublished observation). Thus, these data might indicate that Thoc5 controls global gene expression patterns through the modulation of the fate of different mRNAs. In addition, the human orthologues of mouse Thoc5 targets (e.g. *ENY2*, *GIGYF2* and *SLC11A2*) (37) also display a shortening of the 3′-UTRs on Thoc5 and CFIm68 depletion (Supplementary Table S5 and data not shown). Therefore, the observed developmental defects of Thoc5 knockout mouse embryos (67) reflect a failure to lengthen the 3′-UTRs of some mRNAs because of the lack of Thoc5 activity. Metazoan THO/TREX has also been implicated in tumorigenesis (68), and it would be of interest to examine whether the regulation of 3′-UTR length by Thoc5 is relevant for other biological processes, such as cell proliferation.

## SUPPLEMENTARY DATA

Supplementary Data are available at NAR Online: Supplementary Tables 1–9, Supplementary Figure 1–4 and Supplementary References [14,44].

## FUNDING

Grant-in-Aid for Scientific Research on Innovative Areas ‘RNA regulation’ [20112006 in part] from the Ministry of Education, Culture, Sports, Science and Technology of Japan (MEXT); Grant-in-Aid for Scientific Research (C) [21570195 to J.K.] from the Japan Society for Promotion of Science (JSPS). Funding for open access charge: MEXT and JSPS.

*Conflict of interest statement.* None declared.

## Supplementary Material

Supplementary Data
