# Supplementary material for: Human TREX component Thoc5 affects alternative polyadenylation site choice by recruiting mammalian cleavage factor I
Source: Nucleic Acids Res. 2013 May 17;41(14):7060–72. doi: 10.1093/nar/gkt414 (PMC3737531; doi:10.1093/nar/gkt414)
Supplement: Supplementary Data [file supp_41_14_7060__index.html]

Human TREX component Thoc5 affects alternative polyadenylation site choice by recruiting mammalian cleavage factor I — Human TREX component Thoc5 affects alternative polyadenylation site choice by recruiting mammalian cleavage factor I — Supplementary Data 

# Human TREX component Thoc5 affects alternative polyadenylation site choice by recruiting mammalian cleavage factor I

## Supplementary Data

files

**Files in this Data Supplement:**

- Supplementary Data - pdf file
- Supplementary Data - xlsx file
