# Supplementary material for: Human TREX component Thoc5 affects alternative polyadenylation site choice by recruiting mammalian cleavage factor I
Source: Nucleic Acids Res. 2013 May 17;41(14):7060–72. doi: 10.1093/nar/gkt414 (PMC3737531; doi:10.1093/nar/gkt414)
Supplement: Supplementary Data [file supp_gkt414_nar-00201-r-2013-File003.pdf]

Supplementary Data

Human TREX Component Thoc5 Affects Alternative Polyadenylation Site Choice by Recruiting Mammalian Cleavage Factor I

Jun Katahira[1,2,*], Daisuke Okuzaki[3], Hitomi Inoue[1], Yoshihiro Yoneda[1,2], Kazumitsu Maehara[4], and Yasuyuki Ohkawa[4]

Supplementary Figure legends

Figure S1. Identification of Thoc5 target genes. **(A)** Summary of the microarray analysis. See details in *Materials and Methods*. **(B)** The positions of the probe sets and alternative polyadenylation sites in the *CREB1* gene. The thin lines indicate introns, and the black boxes indicate exons. Both the 5'- and 3'-UTRs are indicated by thinner boxes. The thick lines below the schematic of the *CREB1* gene indicate the positions of the different probe sets. The probe set 214513_s_at shown in red is the proximal probe for this gene; those drawn in black are distal probe sets. The average signal intensities at these probe positions were used to calculate distal/proximal ratios. The green vertical arrows indicate the positions of the reported polyadenylation sites. P, proximal polyadenylation site; D1 to D3, distal polyadenylation sites. Note that the structures of the genes, probe positions, and polyadenylation sites are not drawn to scale.

Figure S2. (A) Scatter plot showing the genes down-regulated at least 2-fold by the indicated siRNA treatment. Total RNAs extracted from siRNA-treated HeLa cells were subjected to expression array analysis. Two-dimensional scatter plot analysis of the simple comparison between the siThoc5- or siCFim68-treated cells (vertical axis) versus siDsRed-treated cells (horizontal axis) was performed using DNA microarray viewer software (Kurabo). Each dot represents the expression values of the different probe sets that were down-regulated at least 2-fold compared with the siDsRed-treated cells showing “Present” calls. The two parallel green and red lines indicate the limits for 2- and 4-fold differences, respectively. **(B)** Probe sets differentially expressed (>2.0-fold change, *p*-value<0.2, see Supplementary Table 1-4 for additional details) in siThoc5- (Bioset 1: BS1) and siCFIm68-treated (Bioset 2: BS2) cells were subjected to NextBio analysis. A Venn diagram shows the number of common and unique probe sets in both experiments. The bar charts at right illustrate the significance of the overlap between the probe subsets. The scale of bar is indicated in –log (*p*-value). Thus, a positive correlation indicates that both features increase or decrease together, whereas a negative correlation indicates that as one features increases, the other feature decreases. Note that strong correlations were observed between the two biosets.

Figure S3. (A) The distributions of CFIm68, Serine2 phosphorylated RNAPII (Ser2-P RNAPII), and TBP on all annotated human genes. The abundance of CFIm68 binding in the siDsRed- samples was plotted along the length of the genes, and the peak information for Ser2-P RNAPII and TBP obtained from the ENCODE project was overlaid. The data are plotted as indicated in Figure 4A. Note that the CFIm68 peak at the 5' regions of the genes almost completely overlapped with those of Ser2-P RNAPII and TBP. **(B)** Distribution of CstF64 and Xrn2 on all annotated human genes. Note that CstF64 and, to a lesser extent Xrn2, exhibit bimodal localization at the 5' and 3' regions of genes. **(C)** The distribution of CstF64 and Xrn2 on the *MYC* and *CDKN1A* genes. The numbers of ChIP-seq reads are plotted on the genomic regions (black bar charts). The peaks for CFIm68 binding were identified using the MACS peak-finding algorithm and indicated using black horizontal bars (CstF64 and Xrn2 peaks). The gene structures are indicated at the bottom of each graph. The horizontal arrows below the gene structures indicate the orientations of the genes. The boxes indicate exons, whereas the thin lines indicate introns. The protein-coding regions are indicated with thicker boxes. Note that CstF64 and Xrn2 peaks were detectable on both side of the genes.

Figure S4. The siRNAs used in this study did not affect the expression of CFIm59.

HeLa cells were treated with the indicated siRNAs for 72 hrs. Whole cell extracts were prepared from each culture and subjected to Western blotting using the antibodies indicated to the right of each panel. The anti-CFIm68 antibody (1) was used to detect the large subunits of CFIm. To visualize the different CFIm large subunits, both shorter (upper panel) and longer (lower panel) exposures of the same blot are shown, and the positions of CFIm68, CFIm72, and CFIm59 are indicated with arrows.

Supplementary Table S1-S4. Significantly altered probe sets upon Thoc5 or CFIm68 knockdown as identified by expression microarray analysis (fold-change > 2). These data are available online as separate Excel sheets.

Supplementary Table S5. Human Thoc5 target genes. This table is available online as a separate Excel sheet.

Supplementary Table S6. ChIP-seq libraries

Name (siRNA/ antibody)	ID in DDBJ database	Mapping rate (%)	Uniquely mapped reads
siDsRed/Input	DRX002709	78.25	19,567,654
siDsRed/CFIm68	DRX002710	62.33	14,303,537
siThoc5/Input	DRX002711	77.78	17,314,754
siThoc5/CFIm68	DRX002712	67.01	20,088,493

Supplementary Table S7. PCR primers used for plasmid construction

---

Probe templates for Northern analysis

RNMT

5'-gacttctgtctgtaggcaagtagacatagc-3'

5'-ttcctgaagttcattgtaatgggcagccac-3'

SUB1

5'-cgagcgaagcgatgcctaaatcaaaggaac-3'

5'-ttacagttttcttactgcatcatcaatgtc-3'

TMED10

5'-ccatctgcccattaactctcgcaagtgcc-3'

5'-taacaatagattctgaaaggtcttctaggc-3'

TIMP2

5'-cccgcaacaggcgttttgcaatgcag-3'

5'-gtgcccgttgatgttcttctctgtgacc-3'

NUDT21

5'-ccgctagcggatccgccatgtctgtggtaccgcccaatcgctcgcag-3'

5'-ggctcgaggaattcagttgtaaataaaattgaacctgctcaacagc-3'

RPL22

5'- ccatggctcctgtgaaaaagcttgtgg-3'

5'- ctggaagtaacgtaattcgtaactctc-3'

Probe templates for RPA

TMED10 proximal pA

5'-ccatctcttaaaatggtgatggatgtgacacc-3'

5'-gcagtgcaagggatccatgtgacagaactg-3'

TMED10 distal pA

5'-ccttaagtaagccttgttataccattgtcatggac-3'

5'-gctcatataaactatcgatctgcagtaactg-3'

TIMP2 proximal pA

5'-gtttctcgacatcgaggacccataagcagg-3'

5'-aggatcgaagccccagacacatagtgcctg-3'

TIMP2 distal pA

5'-acacaagagttgttgaaagttgacaagcag-3'

5'-ggagaggctccttgcagaggctgattcccattcttgagc-3'

Supplementary Table S8. PCR primers used in ChIP PCR

TIMP2

5’- cttctcgaatcctgcaagtggac-3’

5’- ctggagctgcggcggaatttcgg-3’

NUCKS1

5’- tctttctctccgactagaaatgtcc-3’

5’- ggacaagagggttacaaaaatatcc-3’

RPL22

5’- gagaggacgcgttttgcattcagg-3’

5’- tccaggccgcactaatcactttgc-3’

SUB1

5’- gggaagaacggctgatgtgcag-3’

5’- aggggagacaggttggtcggag-3’

*Control (intergenic region in human Chr. 2)

5’- gccttaaggtttataccaaaatca -3’

5’- ggaaggcactgttaaagttgag-3’

* (2)

Supplementary Table S9. Sequences of siRNAs

siCFIm68-1

5’-agacuuaacugaagcaguudTdT-3’

5’-aacugcuucaguuaagucudTdT-3’

siCFIm68-2

5’-agauugccuucauggaauudTdT-3’

5’-aauuccaugaaggcaaucudTdT-3’

SUPPLEMENTARY REFERENCES

1. Ruegsegger,U., Blank,D. and Keller,W. (1998) Human pre-mRNA cleavage factor Im is related to spliceosomal SR proteins and can be reconstituted in vitro from recombinant subunits. *Mol. Cell*, **1**, 243-253.

2. Yamada,T., Yamaguchi,Y., Inukai,N., Okamoto,S., Mura,T. and Handa,H. (2006) P-TEFb-mediated phosphorylation of hSpt5 C-terminal repeats is critical for processive transcription elongation. *Mol. Cell*, **21**, 227-237.

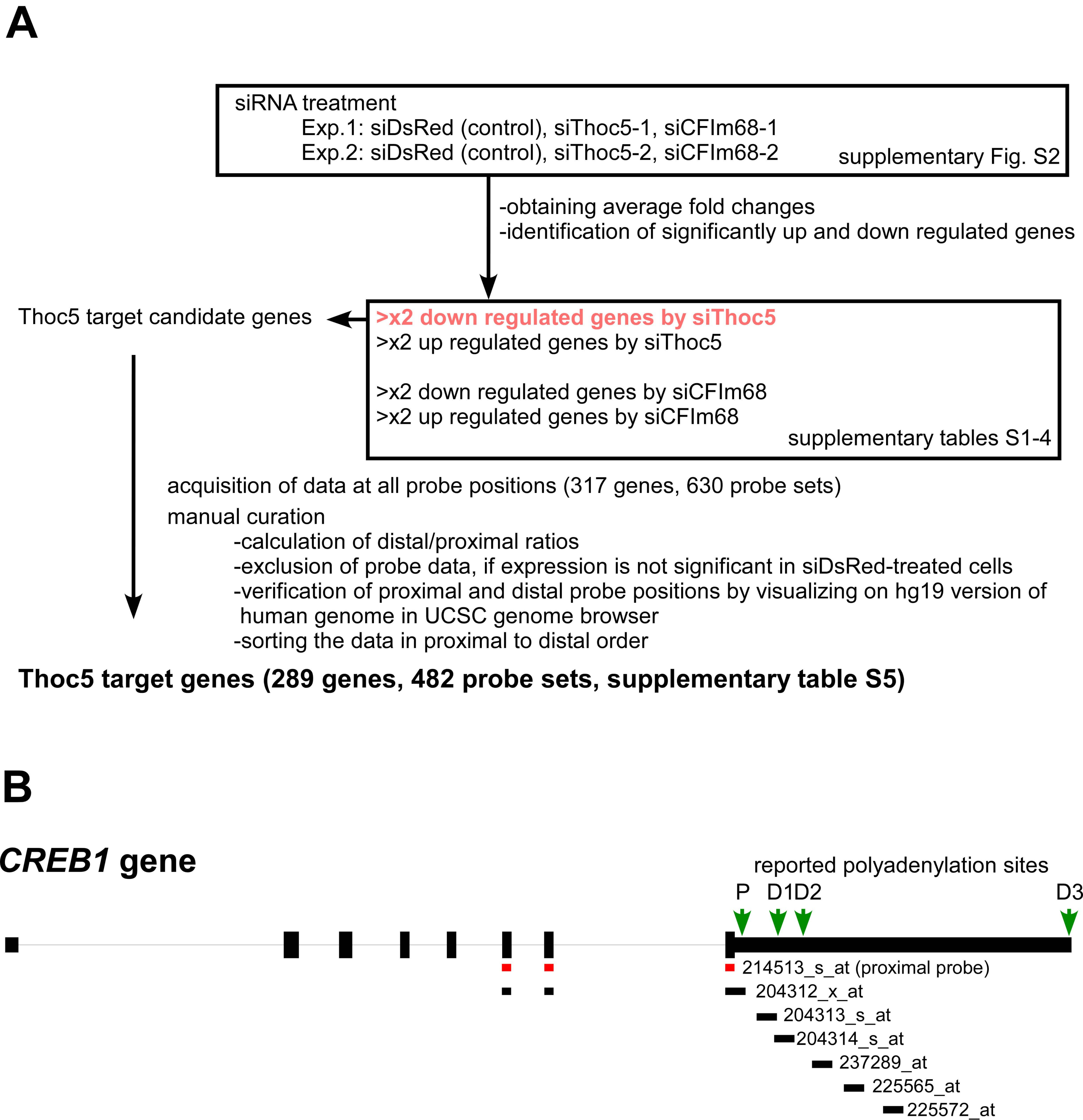
A
siRNA treatment
Exp.1: siDsRed (control), siThoc5-1, siCFIm68-1
Exp.2: siDsRed (control), siThoc5-2, siCFIm68-2
supplementary Fig. S2
-obtaining average fold changes
-identification of significantly up and down regulated genes
Thoc5 target candidate genes
>x2 down regulated genes by siThoc5
>x2 up regulated genes by siThoc5
>x2 down regulated genes by siCFIm68
>x2 up regulated genes by siCFIm68
supplementary tables S1-4
acquisition of data at all probe positions (317 genes, 630 probe sets)
manual curation
-calculation of distal/proximal ratios
-exclusion of probe data, if expression is not significant in siDsRed-treated cells
-verification of proximal and distal probe positions by visualizing on hg19 version of human genome in UCSC genome browser
-sorting the data in proximal to distal order
Thoc5 target genes (289 genes, 482 probe sets, supplementary table S5)
B
CREB1 gene
reported polyadenylation sites
P
D1
D2
D3
214513_s_at (proximal probe)
204312_x_at
204313_s_at
204314_s_at
237289_at
225565_at
225572_at

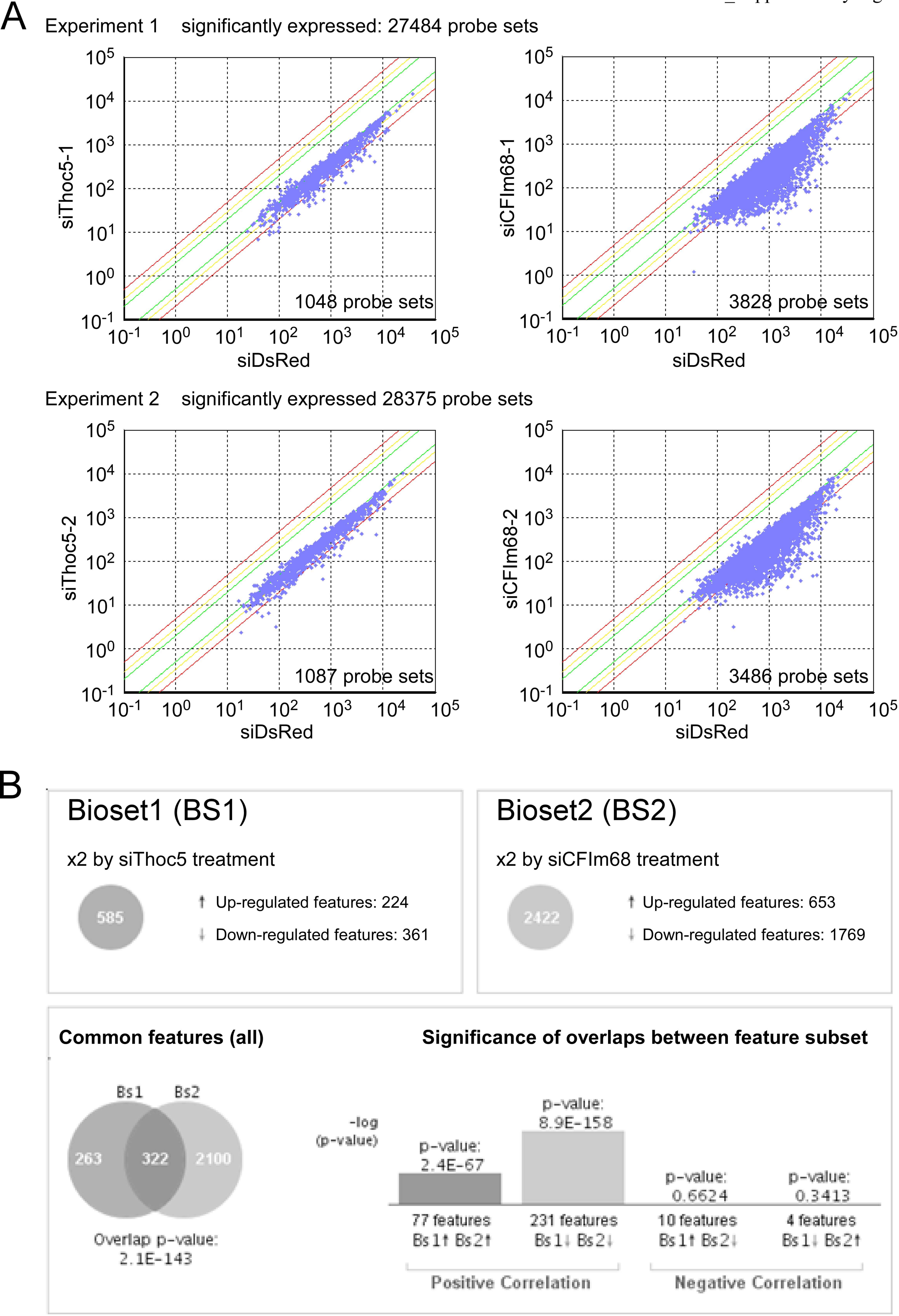
A
Experiment 1 significantly expressed: 27484 probe sets
siThoc5-1
siDsRed
1048 probe sets
siCFIm68-1
siDsRed
3828 probe sets
Experiment 2 significantly expressed 28375 probe sets
siThoc5-2
siDsRed
1087 probe sets
siCFIm68-2
siDsRed
3486 probe sets
B
Bioset1 (BS1)
x2 by siThoc5 treatment
585
Up-regulated features: 224
Down-regulated features: 361
Bioset2 (BS2)
x2 by siCFIm68 treatment
2422
Up-regulated features: 653
Down-regulated features: 1769
Common features (all)
Bs1
Bs2
263
322
2100
Overlap p-value: 2.1E-143
Significance of overlaps between feature subset
-log (p-value)
p-value: 2.4E-67
p-value: 8.9E-158
p-value: 0.6624
p-value: 0.3413
77 features Bs1↑ Bs2↑
231 features Bs1↓ Bs2↓
10 features Bs1↑ Bs2↓
4 features Bs1↓ Bs2↑
Positive Correlation
Negative Correlation

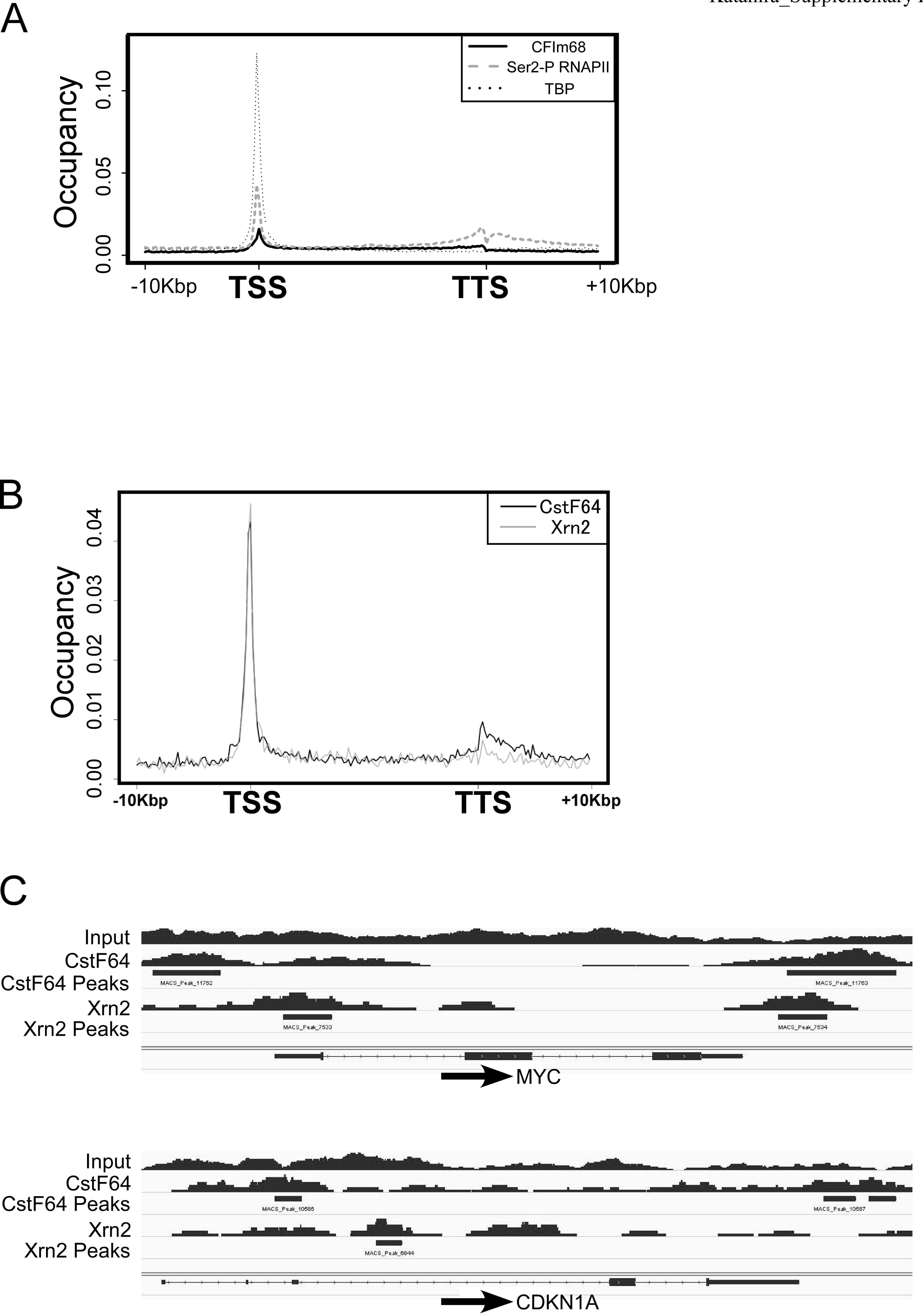
A
CFIm68
Ser2-P RNAPII
TBP
Occupancy
0.00
0.05
0.10
-10Kbp
TSS
TTS
+10Kbp
B
CstF64
Xrn2
Occupancy
0.00
0.01
0.02
0.03
0.04
-10Kbp
TSS
TTS
+10Kbp
C
Input
CstF64
CstF64 Peaks
Xrn2
Xrn2 Peaks
MACS_Peak_11762
MACS_Peak_11763
MACS_Peak_7533
MACS_Peak_7534
MYC
Input
CstF64
CstF64 Peaks
Xrn2
Xrn2 Peaks
MACS_Peak_10586
MACS_Peak_10587
MACS_Peak_6844
CDKN1A

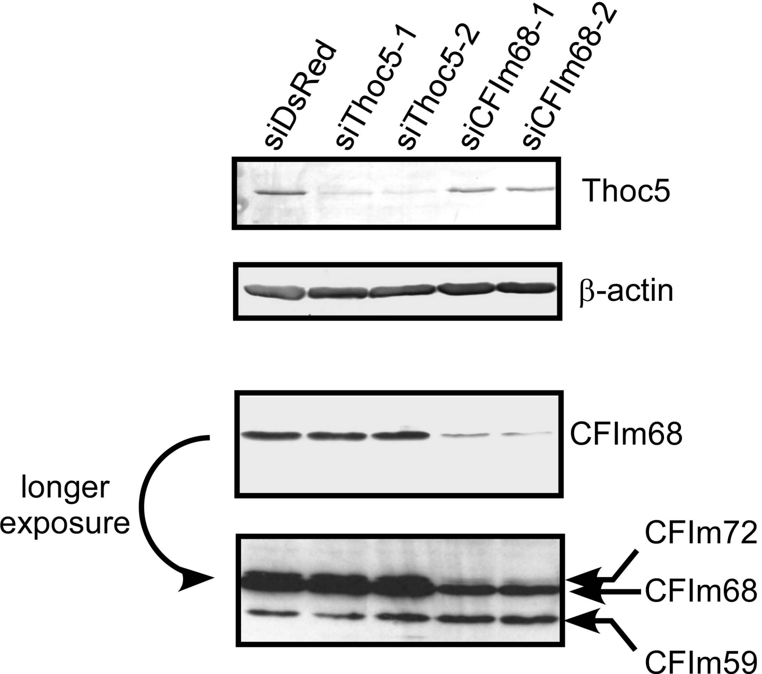
siDsRed
siThoc5-1
siThoc5-2
siCFIm68-1
siCFIm68-2
Thoc5
β-actin
CFIm68
longer exposure
CFIm72
CFIm68
CFIm59
